# Hydrogen-rich water treatment increased several phytohormones and prolonged the shelf life in postharvest okras

**DOI:** 10.3389/fpls.2023.1108515

**Published:** 2023-02-14

**Authors:** Wanqi Dong, Shifeng Cao, Qihang Zhou, Shuwan Jin, Chujiang Zhou, Qingli Liu, Xu Li, Wei Chen, Zhenfeng Yang, Liyu Shi

**Affiliations:** College of Biological and Environmental Sciences, Zhejiang Wanli University, Ningbo, China

**Keywords:** okra, hydrogen-rich water, melatonin, indoleacetic acid, gibberellin, abscisic acid

## Abstract

Hydrogen-rich water (HRW) treatment has been reported to delay the softening and senescence of postharvest okras, but its regulatory mechanism remains unclear. In this paper, we investigated the effects of HRW treatment on the metabolism of several phytohormones in postharvest okras, which act as regulatory molecules in fruit ripening and senescence processes. The results showed that HRW treatment delayed okra senescence and maintained fruit quality during storage. The treatment upregulated all of the melatonin biosynthetic genes such as *AeTDC*, *AeSNAT*, *AeCOMT* and *AeT5H*, contributing to the higher melatonin content in the treated okras. Meanwhile, increased transcripts of anabolic genes but lower expression of catabolic genes involved in indoleacetic acid (IAA) and gibberellin (GA) metabolism were observed in okras when treated with HRW, which was related to the enhanced levels of IAA and GA. However, the treated okras experienced lower abscisic acid (ABA) content as compared to the non-treated fruit due to the down-regulation of its biosynthetic genes and up-regulation of the degradative gene *AeCYP707A*. Additionally, there was no difference in γ-aminobutyric acid between the non-treated and HRW-treated okras. Collectively, our results indicated that HRW treatment increased levels of melatonin, GA and IAA, but decreased ABA content, which ultimately delayed fruit senescence and prolonged shelf life in postharvest okras.

## Introduction

Horticulture is a dynamic sector where a wide variety of crops and their products are continuously innovating, and new market opportunities are considered and explored. It includes the cultivation of fruits, vegetables, nuts, seeds, herbs, sprouts, mushrooms, algae, flowers, seaweeds, and non-food crops such as grass and ornamental trees and plants. However, this sector faces many challenges, from retaining economic competitiveness, achieving full economic and environment sustainability and responding to climate change (Fawad et al., 2021; Taskesenlioglu et al., 2022). In addition, growing population and rise in income level will lead to increase in demand of high-value agriculture (HVA) produce (Ghosh, 2012).

Okra (*Abelmoschus esculentus* L.) as a vegetable, is now becoming more and more popular in China because of its rich nutritive value (Petropoulos et al., 2018). Generally, okra is extremely vulnerable to mechanical damage and therefore quite difficult for storage after harvest. According to statistics, the loss rate of postharvest okra ranges from 50% to72% (Gajanan et al., 2018). Therefore, it is of great importance to develop its preservation methods and prolong its shelf life.

In recent years, the role of hydrogen against abiotic stress in plants has been widely studied (Wu et al., 2019; Yan et al., 2022; Yu et al., 2023). In addition, hydrogen also plays an important role in maintaining fruit quality and delaying senescence process in several postharvest vegetables and fruit. For instance, 25% hydrogen-rich water (HRW) treatment improved the activities of antioxidant enzymes and inhibited the decay incidence of mushrooms (Chen et al., 2017). HRW treatment also increased fruit quality through regulating antioxidant capacity and energy metabolism in Rosa sterile fruit (Dong et al., 2022).

Plant hormones are chemical compounds present in very low concentration in plant, appearing to be multi-regulatory molecules during fruit development, ripening and senescence (Poveda, 2020). Indolacetic acid (IAA), which exists in almost all plant organs, is the main form of plant auxin (Yue et al., 2019). It was reported that IAA content accumulated during fruit ripening but decreased rapidly during fruit senescence (Trainotti et al., 2007; Chen et al., 2014). Exogenous treatment with IAA could delay the ripening process in harvested kiwifruit (Gan et al., 2021). The protective effects of melatonin as an antioxidant has been frequently investigated (Reiter et al., 2001; Iriti et al., 2006). Its effect on prolonging shelf life and improving fruit quality has been demonstrated in postharvest strawberries and table grapes (Liu et al., 2018; Sun et al., 2020). Abscisic acid (ABA) has a key role in regulating fruit senescence (McAtee et al., 2013) and conferring tolerance to environmental stresses. Previous studies have showed that ABA induced fruit senescence in postharvest sweet cherries (Luo et al., 2014) and enhanced the resistance against chilling in harvested zucchini (Carvajal et al., 2017). A tetracyclic diterpenoid hormone known as gibberellic acid (GA) has been reported to maintain fruit quality and prolong shelf life in postharvest okras (Xiao et al., 2022), Chinese flowering cabbage (Fan et al., 2021), persimmons (Maurer et al., 2019).

In a previous study, postharvest okra softening was delayed and storage life was prolonged by treatment with 0.22 mM HRW (Dong et al., 2023). However, there is no information available on the effect of hydrogen (H_2_), as an important signal molecule, on the regulation of metabolism of plant hormones in postharvest fruit or vegetables. Therefore, in order to further understand the mechanism of H_2_ in the preservation of okras after harvest, here we determined the levels of several hormones such as melatonin, IAA, ABA and GA3 together with their metabolizing genes in okras treated with HRW to reveal the relationship between H_2_ and other signal molecules.

## Materials and methods

### Hydrogen-rich water preparation, okra treatment and storage

HRW was prepared as the protocol of Dong et al. (2023). Fresh okras were purchased from a farm in Fenghua, Ningbo, China. The sample of the same size and maturity, free from disease and mechanical damage were selected. Okras were randomly divided into two experimental groups with two hundred okras in each. The HRW treatment was same as our previous study (Dong et al., 2023). After treatment, all the okras were surface-dried and packed in 0.03 mm polyethylene bags (5 mm × 5 mm diameter holes per bag, 10 fruit per bag). Then The fruit were stored in a constant temperature and humidity incubator at 25 ± 1°C and 80% ~ 90% relative humidity for 12 d. Thirty okras were taken from the control and treatment groups every 3 d for analysis.

### Determination of senescence index and ΔE value

Senescence index manifested as browning discoloration and appearance on the surface of ten okras from each replicate were evaluated visually. For each okra, senescence was scored based on a five-grade system, where 0 means no shrinkage, no browning, mildew and bright color; 1 means slight shrinkage and browning but no mildew; 2 means obvious shrinkage, browning, mildew and yellowing; 3 means severe shrinkage, browning and mildew, yellow and black color with pungent odor; 4 means rotten into mud with strong pungent odor. The results were expressed as a senescence index calculated according to the formula below: Senescence index (between 0 and 4) = [(senescence level) * (number of okras at the senescence level)]/(total number of fruit *4) *100%. After measuring the L^*^, a^*^, b^*^ values of okras with a colorimeter (KONICA MINOLTA, Japan), the ΔE value was calculated using the following formula: ΔE^*^_ab_=∑ $\sqrt{{\left(L_{2}^{*}-L_{1}^{*}\right)}^{2}-{\left(a_{2}^{*}-a_{1}^{*}\right)}^{2}-{\left(b_{2}^{*}-b_{1}^{*}\right)}^{2}}$ , where *L*_1_^*^/*a*_1_^*^/*b*_1_^*^= measured value of sample before treatment; *L*_2_^*^/*a*_2_^*^/*b*_2_^*^= measured value of sample at the sampling point.

### Determination of melatonin, abscisic acid, indolacetic acid, gibberellin and γ-aminobutyric acid levels

Melatonin, ABA, IAA and GA levels were determined using corresponding commercially ELISA kits under instruction (Jiangsu Meimian industrial Co., Ltd, Nanjing, China). GABA was extracted and determined using Wang’s methods (2019).

### Gene expression analysis

Extraction and reverse transcription of total RNA were performed according to the method of Dong et al. (2023). Gene expression analysis was performed on a StepOnePlus™ real-time PCR instrument (BIO-RAD, Hercules, California, USA) using SYBR Green I Master Mix (Vazyme, Nanjing, Jiangsu, China) and specific primers (**Supplementary Table 1**). Gene expression was normalized to *AeACT* and calculated using the 2^-ΔCt^ method.

### Statistical analysis

GraphPad Prism 9 was used to analyze the experimental data. Differences between control and treatment were tested using multiple t tests (* *P*< 0.05, ** *P*< 0.01, and *** *P*< 0.001).

## Results

### Effects of hydrogen-rich water treatment on shelf life of postharvest okra

**Figure 1:**
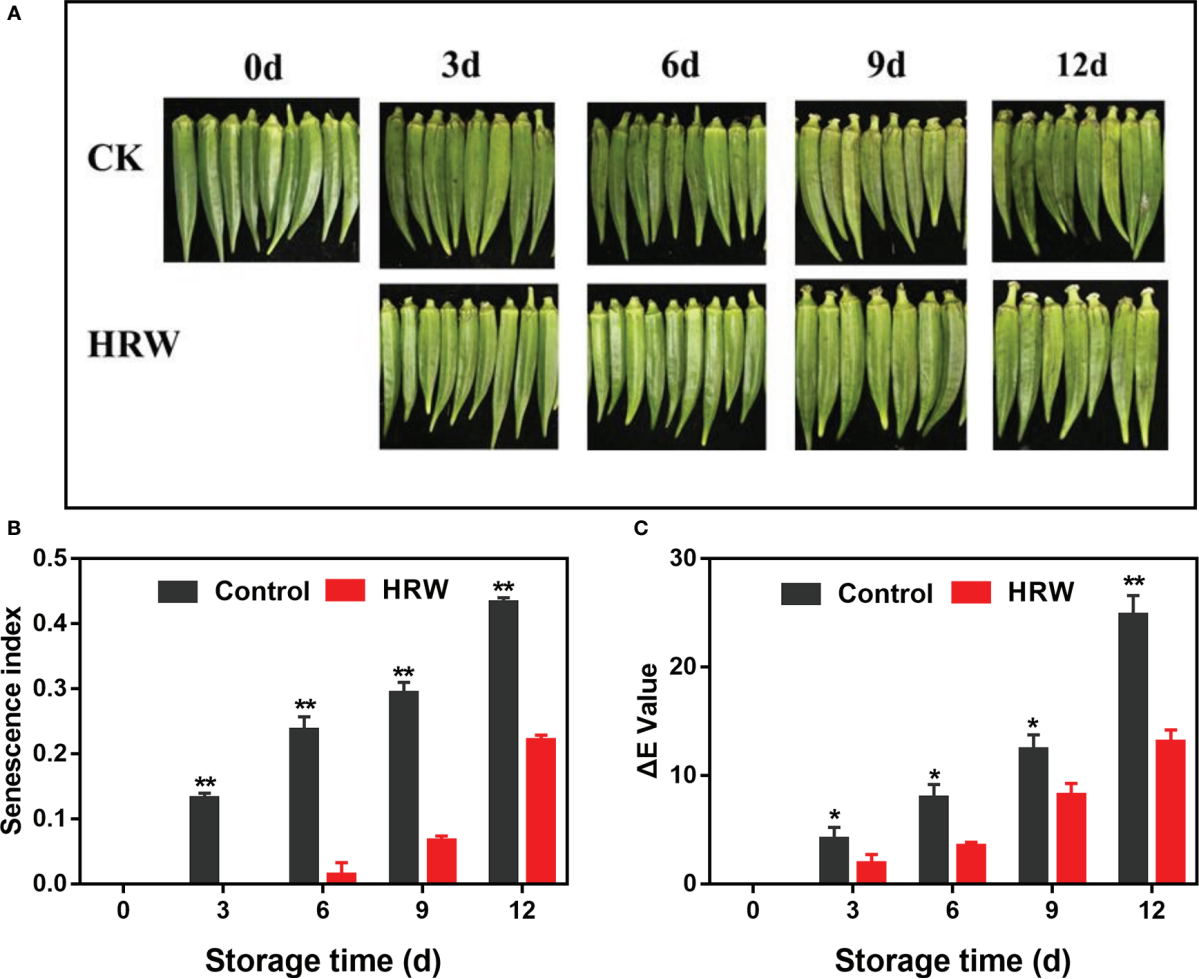
Hydrogen-rich water treatment affected appearance **(A)**, senescence index **(B)** and ΔE value **(C)** of okras stored at 25°C. Asterisks indicate significant differences between Control and HRW treatment (*p < 0.05, **p < 0.01, and ***p < 0.001).

The controlled okras slowly became shrivelled and tarnished during storage, together with the gradual increase in senescence index and ΔE values. However, HRW treatment delayed okra senescence and maintained fruit quality during storage (**Figure 1**).

### Effects of HRW treatment on melatonin content and expression of biosynthesis-related genes in okra during storage

**Figure 2:**
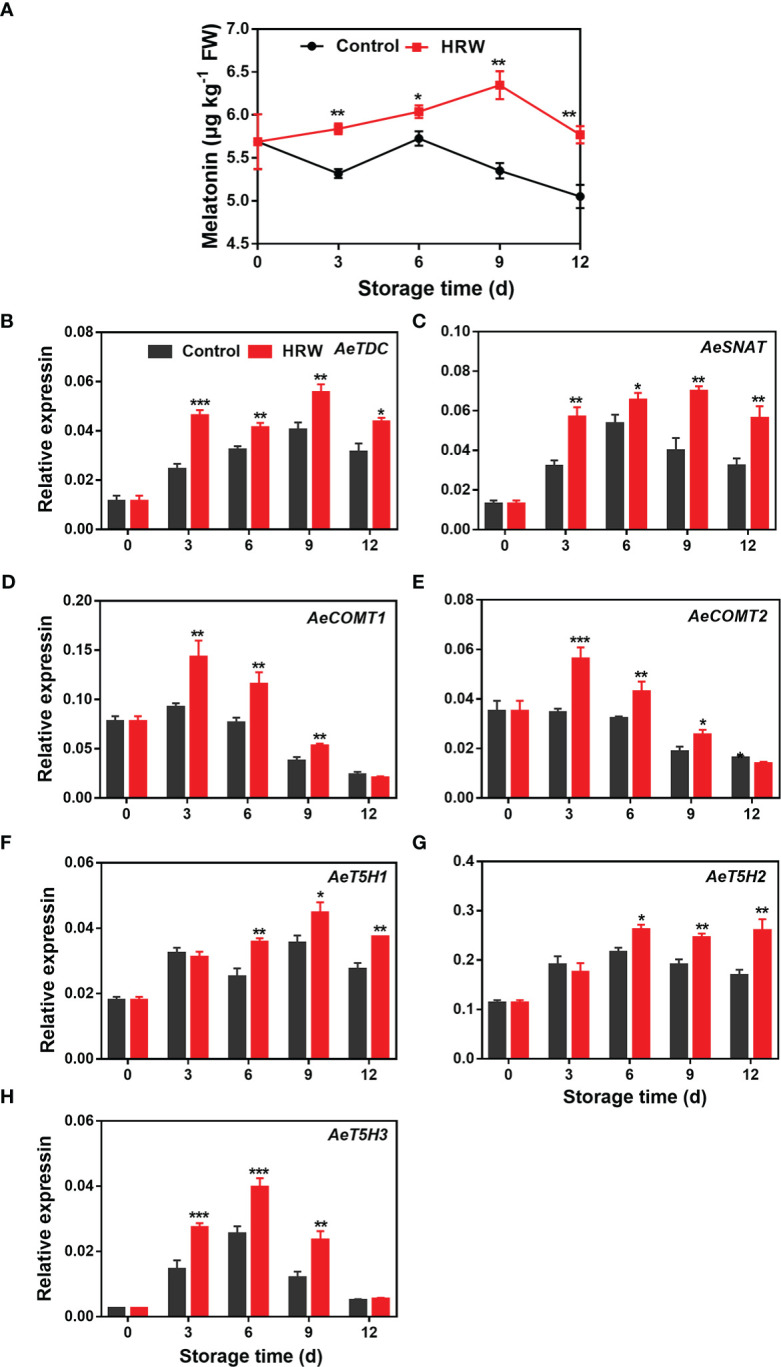
Hydrogen-rich water treatment affected melatonin content **(A)** and gene expression of *AeTDC* **(B)**, *AeSNAT* **(C)**, *AeCOMT1* **(D)**, *AeCOMT2* **(E)**, *AeT5H1* **(F)**, *AeT5H2* **(G)**, *AeT5H3* **(H)** of okras stored at 25°C. Asterisks indicate significant differences between Control and HRW treatment (*p < 0.05, **p < 0.01, and ***p < 0.001).

The melatonin contents in both HRW- and non-treated okras increased firstly followed by a decline thereafter. HRW treatment increased its content in okra throughout the storage (**Figure 2A**). At the end of storage, the content was 14.2% higher than the untreated okras. Correspondingly, all of the melatonin biosynthetic genes investigated here exhibited an increasing trend firstly and then declined during the remaining storage time. HRW treatment increased the expressions of *AeTDC* and *AeSNAT* during the whole storage (**Figures 2B, C**). Higher transcripts of *AeCOMT1/2* were observed in treated okras on day 3, 6 and 9 (**Figures 2D, E**). Meanwhile, the treatment upregulated the expression of *AeT5H1/2* after 6 d and *AeT5H3* on day 3, 6 and 9 of storage (**Figures 2F–H**).

### Effects of HRW treatment on gibberellin content and expression of gibberellin metabolizing genes in okra during storage

**Figure 3:**
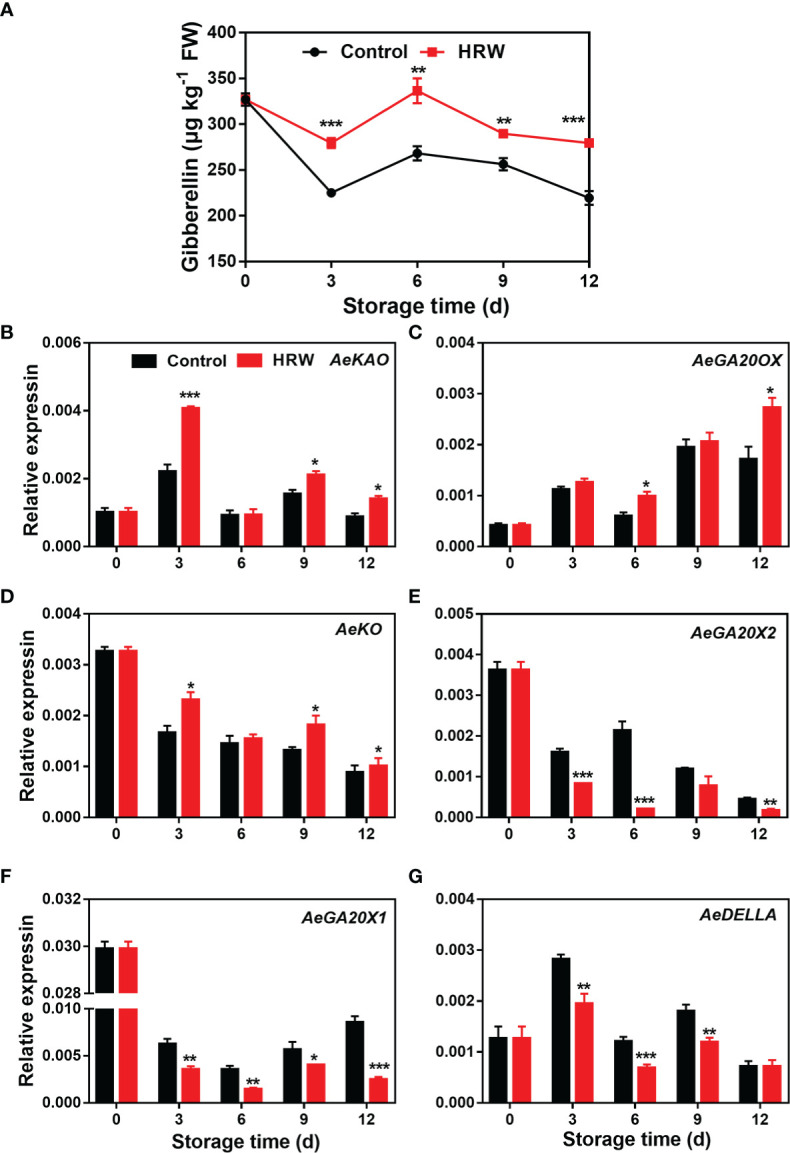
Hydrogen-rich water treatment affected gibberellin content **(A)** and gene expression of *AeKAO* **(B)**, *AeGA20OX* **(C)**, *AeKO* **(D)**, AeGA20X2 **(E)**, *AeGA20X1* **(F)**, *AeDELLA* **(G)** of okras stored at 25°C. Asterisks indicate significant differences between Control and HRW treatment (*p < 0.05, **p < 0.01, and ***p < 0.001).

As okras were stored for the first six days, the GA contents increased, followed by a decline thereafter. Higher content of GA was observed in okras after HRW treatment throughout the whole storage (**Figure 3A**). As compared to the non-treated okras, higher transcripts of *AeKAO* and *AeKO* were observed on day 3, 9 and 12 of storage and *AeGA20OX* on day 6 and 12 in fruit with HRW treatment (**Figures 3B–D**). The expression levels of *AeGA20X1* and *AeGA20X2* declined firstly in both control and treated okras. Their expression was downregulated throughout the storage by the treatment (**Figures 3E, F**). Meanwhile, *AeDELLA* expression was also down-regulated by HRW on day 3, 6, and 9 (**Figure 3G**).

### Effects of HRW treatment on indolacetic acid content and expression of indolacetic acid metabolizing genes in okra during storage

**Figure 4:**
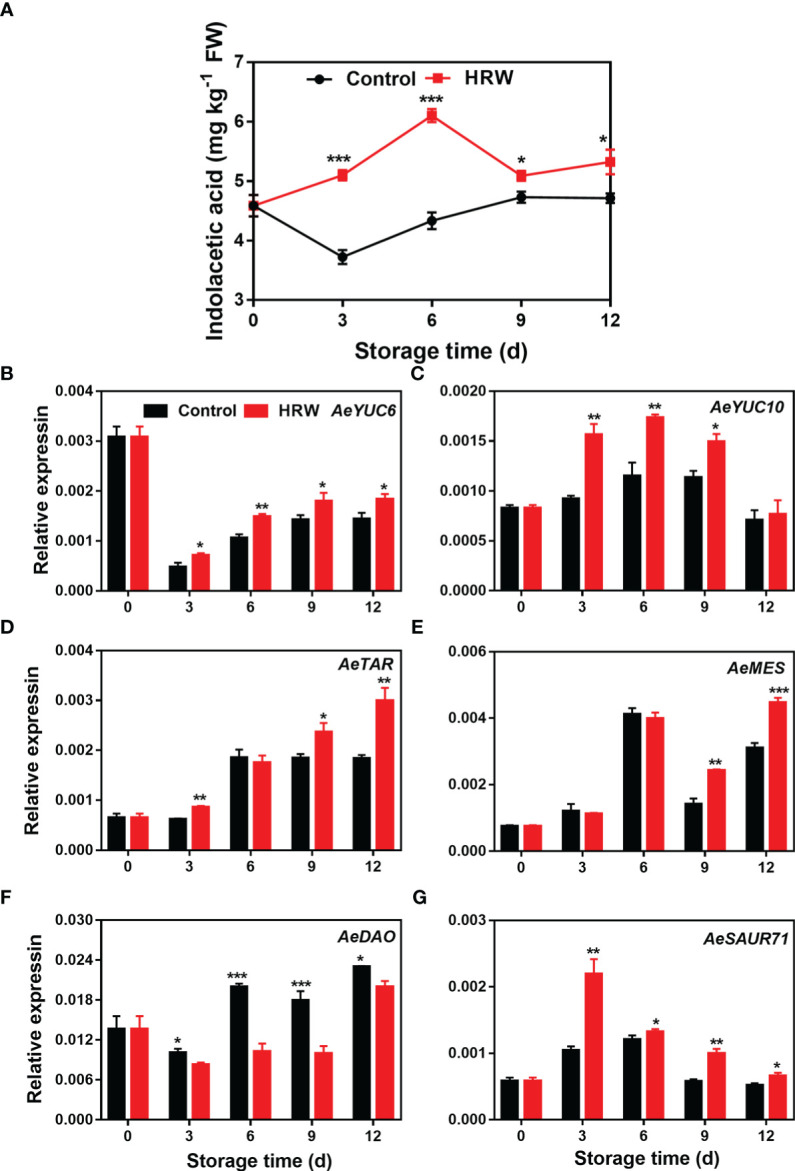
Hydrogen-rich water treatment affected indolacetic acid content **(A)** and gene expression of *AeYUC6* **(B)**, *AeYUC10* **(C)**, *AeTAR* **(D)**, *AeMES* **(E)**, *AeDAO* **(F)**, *AeSAUR71* **(G)** of okras stored at 25°C. Asterisks indicate significant differences between Control and HRW treatment (*p < 0.05, **p < 0.01, and ***p < 0.001).

After HRW treatment, IAA content in okras increased rapidly during the first 6 d of storage. However, the level of IAA in control fruit declined firstly then increased slightly afterwards. IAA content in the treated-okras was higher than that in the controls (**Figure 4A**). In the first 3 d of storage, *AeYUC6* transcript abundance decreased drastically and then continued to increase. HRW treatment increased its expression during storage (**Figure 4B**). In control okras, *AeYUC10* and *AeTAR* expression initially increased, then declined. Compared to the untreated- okras, fruit treated with HRW had higher levels of *AeYUC10* on day 3, 6 and 9 (**Figure 4C**). The treatment also upregulated *AeTAR* expression on day 3, 9 and 12 (**Figure 4D**). No significant difference was found in the abundance of *AeMES* transcripts between the control and treated okras during first 6 d of storage, but it was increased by the treatment after that (**Figure 4E**). For the degradative gene *AeDAO*, HRW down-regulated its expression within the storage (**Figure 4F**). *AeSAUR71*’s expression in both control and treated okras initially increased, then declined afterwards. Higher expression of *AeSAUR71* was observed in HRW-treated okras during storage when compared to the control (**Figure 4G**).

### Effects of HRW treatment on abscisic acid content and expression of abscisic acid metabolizing genes in okra during storage

**Figure 5:**
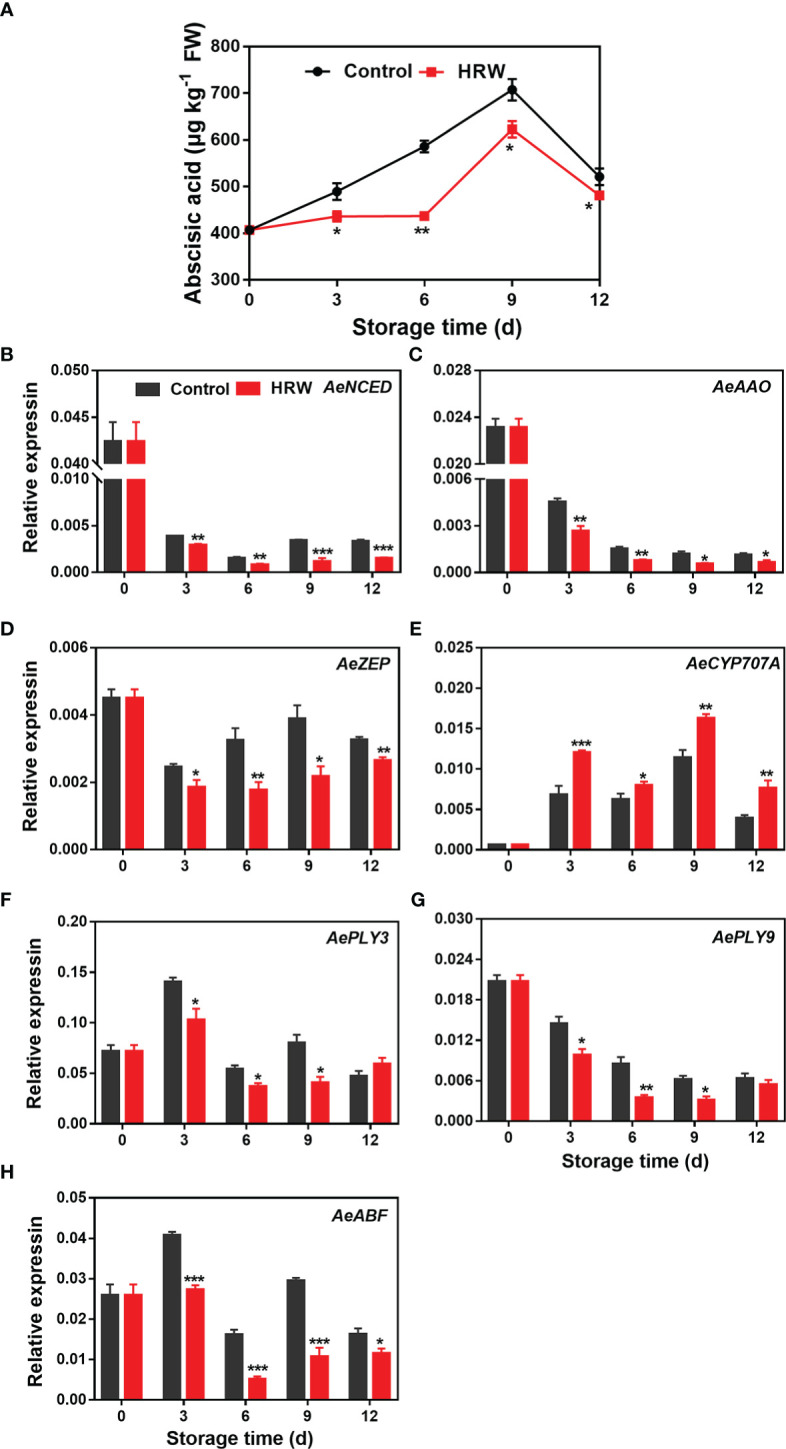
Hydrogen-rich water treatment affected abscisic acid content **(A)** and gene expression of *AeNCED* **(B)**, *AeAAO* **(C)**, *AeZEP* **(D)**, *AeCYP707A* **(E)**, *AePLY3* **(F)**, *AePLY9* **(G)**, *AeABF* **(H)** of okras stored at 25°C. Asterisks indicate significant differences between Control and HRW treatment (*p < 0.05, **p < 0.01, and ***p < 0.001).

ABA content in both HRW- and non-treated okras increased during the first 9 d of storage and then declined towards the end. Lower ABA content was found in HRW-treated okras during the storage (**Figure 5A**). The expression of *AeNCED* and *AeAAO* declined dramatically during the first 3 d of storage, then the level was maintained at a low level. HRW treatment down-regulated these two genes within the storage (**Figures 5B, C**). As compared to the okras treated with HRW, higher transcripts of *AeZEP* and lower ABA degradative gene *AeCYP707A* could be observed in non-treated fruit (**Figures 5D, E**). In addition, HRW treatment also down-regulated the expression of *AePLY3/9* on day 3, 6 and 9 (**Figures 5F, G**) but decreased the transcripts of *AeABF* during the whole storage (**Figure 5H**).

### Effects of HRW treatment on γ-aminobutyric acid content and expression of γ-aminobutyric acid biosynthesis-related genes in okra during storage

**Figure 6:**
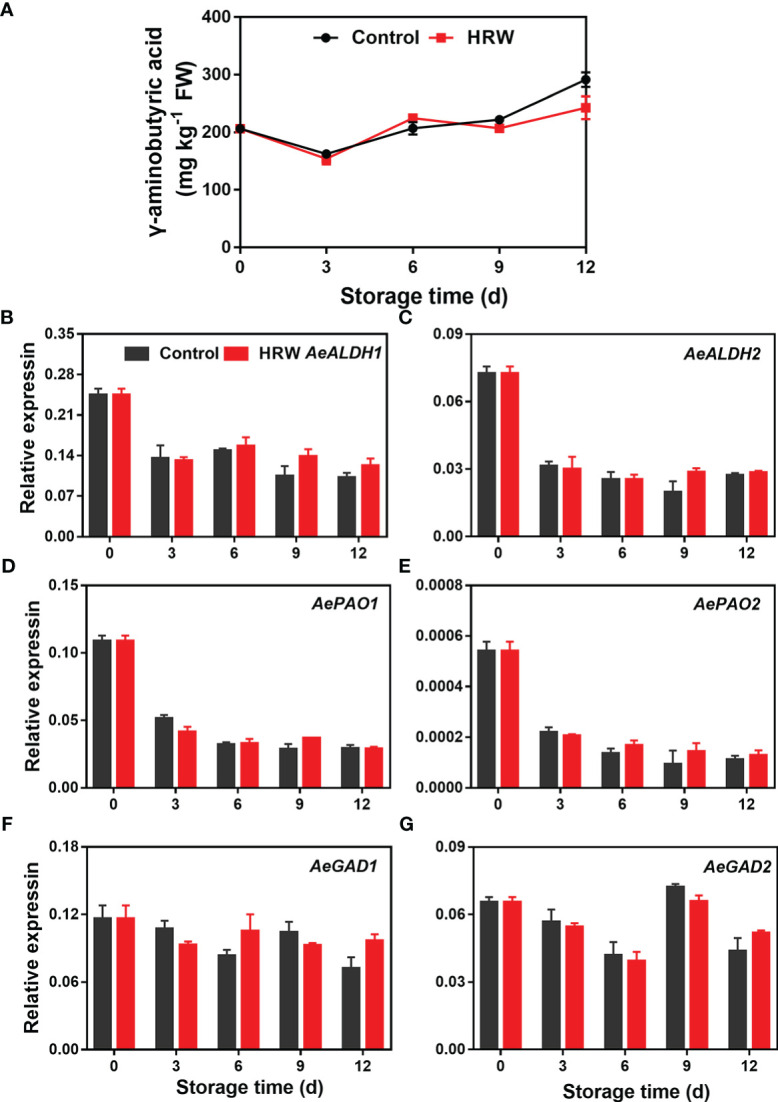
Hydrogen-rich water treatment affected γ-aminobutyric acid content **(A)** and gene expression of *AeALDH1* **(B)**, *AeALDH2* **(C)**, *AePAO1* **(D)**, *AePAO2* **(E)**, *AeGAD1* **(F)**, *AeGAD2* **(G)**, of okras stored at 25°C.

A slight increase of GABA content in both control and the treated okras was observed during storage, there was no significant difference in its content between the non- and treated okras (**Figure 6A**). The expression of *AeALDH1/2* and *AePAO1/2* was observed a significant decline during the first three days of storage, then decreased and remained stable till the end of storage. The expression level of *AeGAD1* and *AeGAD2* in the two groups experienced a similar change during storage. No significant difference in the transcripts of all these six GABA biosynthetic genes was observed between the non- and treated okras (**Figures 6B–G**).

## Discussion

As an important bio-regulator in plants, hydrogen has critical roles in diverse processes in plant growth, development and stress responses (Ren et al., 2017a; Yu et al., 2023). Recently, attention to the role of hydrogen has shifted from plant to postharvest vegetables and fruit. For example, HRW treatment was found to improve antioxidant capacity and extend the shelf life of pakchoi (An et al., 2021). Additionally, kiwifruit and mushrooms treated with HRW showed higher free radical scavenging activity and lower rot rate as compared to the non-treated groups (Chen et al., 2017; Hu et al., 2018). In our previous study, we observed that hydrogen-rich water treatment delayed the softening process and prolonged the storage time by modifying the cell wall metabolism and cell wall composition in postharvest okras (Dong et al., 2023). Similarly, here we demonstrated again that HRW treatment could prolong the shelf life of postharvest okras.

In the last few years, a growing number of studies have revealed how hydrogen interacts with other plant hormone signalling pathways, indicating the key role it plays in protecting plants from stress (Zeng, 2013). H_2_ enhanced the drought resistance of tomato seedlings by regulating the expression of genes in ABA biosynthesis and signal transduction (Yan et al., 2022); H_2_ was also reported to be involved in auxin-induced lateral root formation (Cao et al., 2017). Melatonin, a well-known animal hormone that the brain produces in response to darkness, was first discovered in plants in 1995 (Dubbels et al., 1995), and since then its role in plants has been discovered and studied widely. Accumulating documents over the past decades demonstrated that melatonin could fortify plants against senescence and biotic stresses such as salt, chilling, drought and heavy metals (Cai et al., 2017; Wang et al., 2017; Yan et al., 2019). In addition to inducing stress resistance in plants, much progress has been made recently in understanding the roles of melatonin in regulating storage time in postharvest fruit. Melatonin application enhanced antioxidant defense and increased tolerance to chilling in cold-stored peaches (Cao et al., 2018). The postharvest treatment with melatonin inhibited decay incidence and delayed in strawberries (Liu et al., 2018). Through our experiments, we found that the melatonin content and its biosynthetic genes such as *AeTDC*, *AeT5H*, *AeSNAT* and *AeCOMT* were upregulated in okras after HRW treatment, indicating the increased melatonin biosynthesis in postharvest okras treated with HRW contribution to the delay of senescence and prolongation of shelf life. Furthermore, Su et al. (2021) reported that melatonin acted downstream of molecular hydrogen and its combination with molecular hydrogen induced salt tolerance in Arabidopsis. Therefore, it is reasonable to speculate that the crosstalk between molecular hydrogen and melatonin also plays a role in delaying senescence in postharvest fruit and vegetable.

Lately, Wu et al. (2020) revealed that HRW treatment enhanced the growth of mung bean seedlings by stimulating elongation of hypocotyl and root cells *via* increasing GA and IAA content, suggesting that hydrogen acted upstream of IAA and GA biosynthesis during plant growth. Meanwhile, GA and IAA have been demonstrated their roles in inhibiting the senescence process in postharvest okras and strawberries, respectively (Chen et al., 2014; Xiao et al., 2022). Therefore, to further reveal the crosstalk between hydrogen and GA or IAA, the biosynthetic and signalling pathways of these two phytohormones were investigated in okras after HRW treatment. In our present study, HRW upregulated the anabolic genes such as *AeGA20OX*, *AeKO* and *AeKAO*, but downregulated the catabolic gene *AeGA20X*s and *AeDELLA* the negative regulator of gibberellin signalling, thereby increasing GA content and signalling in postharvest okras. In respect of IAA biosynthesis, the treatment also increased the IAA content along with the enhanced transcripts of biosynthetic genes including *AeTAR*, *AeYUC* and *AeMES* and the early auxin-responsive gene *AeSAUR*, however, the lower expression of the degradative gene *AeDAO* was observed in the treated okras. Moro et al. (2017) found that postharvest exogenous IAA treatment postponed fruit ripening by affecting anthocyanin biosynthesis in red raspberries. It has also been reported that the application of exogenous GA inhibited fruit softening and delayed the loss of ascorbic acid and soluble reducing sugar, thereby prolonging the shelf life of banana fruit (Huang et al., 2014). Recently, in carambola fruit, exogenous 2,4-epibrassinolide treatment maintained fruit quality and inhibited fruit ripening due to the increase in levels of IAA and GA in carambola fruit (Zhu et al., 2021). Therefore, taken together, we could conclude that HRW treatment delayed senescence and extended the shelf life of postharvest okras also due to the increase of levels of GA and IAA.

ABA, another plant hormone, regulates numerous aspects of fruit ripening, senescence and stress responses. It not only enhanced the ripening process of climacteric fruit like kiwifruit and mango (Zaharah et al., 2013; Gan et al., 2020), but also promoted the senescence of non-climacteric fruit such as sweet cherries (Luo et al., 2014). Previous studies have showed that the application of alginate oligosaccharide inhibited the accumulation of ABA and reduced the degradation of cell wall components, thereby maintaining fruit quality in strawberries (Bose et al., 2019). Gan et al. (2020) found silencing *AeNCED1* the key gene in ABA biosynthesis in kiwifruit could decline ABA content and postpone fruit softening. The inhibition of fruit ripening in carambola fruit treated with 2,4-epibrassinolide was also related to the decrease in ABA content (Zhu et al., 2021). Our experimental results showed that HRW treatment reduced the expression of genes related to ABA synthesis such as *AeNCED*, *AeZEP*, and *AeAAO* but increased the transcripts of degradative gene *AeCYP707A*, thus finally reducing ABA content, which was associated with the inhibition of senescence in okras with HRW treatment. It has been reported that both PLYs and ABFs play important roles in regulating Arabidopsis leaf senescence triggered by ABA (Gao et al., 2016; Zhao et al., 2016). In our present study, corresponding to the ABA level, *AePLY3/9* and *AeABF* were also downregulate by HRW treatment, suggesting that hydrogen could regulate ABA-associated signals to slow down the senescence process in postharvest okras.

The role of GABA in fruit ripening, senescence and tolerance against stresses was well reported in different kinds of postharvest fruit and vegetables (Sheng et al., 2017; Palma et al., 2019; Asgarian et al., 2022). However, in our present study, there was no difference in GABA content together with the expression of its metabolizing genes between the control and HRW treated okras, suggesting that no interaction between H_2_ and GABA existed in postharvest okras.

## Conclusion

In conclusion, our data demonstrated that HRW treatment could regulate the contents of several phytohormones and consequently prolong shelf life in postharvest okras. Our results suggested that melatonin, IAA and GA were negatively while ABA was positively associated with fruit senescence in postharvest okras. The increased shelf life and delaying okra senescence by HRW treatment was due to its capability to increase melatonin, IAA and GA contents and inhibit ABA level resulted from its regulation of their metabolizing genes, indicating H_2_, as a signaling molecule, could interrelate to these hormones and influenced their biosynthesis and signaling and finally co-adjusted okra senescence after harvest. However, more evidence is needed to determine the underline cross-talk regulation and transduction relationship between H_2_ and other phytohormones for further exploration on the role of H_2_ in the shelf life prolongation of postharvest products.

## Data availability statement

The original contributions presented in the study are included in the article/**Supplementary Material**. Further inquiries can be directed to the corresponding author.

## Author contributions

WD: Perform the experiment, write the original manuscript. SC and QZ: Conceived and designed the experiment. SJ, CZ and QL: Formal analysis, Investigation. XL and WC: Conducted the experiments, analyzed the data. LS and ZY: Funding acquisition, supervision and manuscript editing. All authors contributed to the article and approved the submitted version.
